# Baricitinib decreases anti-dsDNA in patients with systemic lupus erythematosus: results from a phase II double-blind, randomized, placebo-controlled trial

**DOI:** 10.1186/s13075-022-02794-x

**Published:** 2022-05-16

**Authors:** Thomas Dörner, Ronald F. van Vollenhoven, Andrea Doria, Bochao Jia, Jorge A. Ross Terres, Maria E. Silk, Stephanie de Bono, Peter Fischer, Daniel J. Wallace

**Affiliations:** 1 Department Medicine/Rheumatology and Clinical Immunology, Charite – Universitätsmedizin Berlin and Deutsches Rheuma-Forschungszentrum (DRFZ), Chariteplatz, 01 10117 Berlin, Germany; 2 University Medical Center, Amsterdam, The Netherlands; 3 University of Padova, Padova, Italy; 4 Eli Lilly and Company, Indianapolis, IN USA; 5 Cedars-Sinai Medical Center and University of California at Los Angeles, Los Angeles, CA USA

**Keywords:** Baricitinib, Cytokines, JAK inhibitor, Systemic lupus erythematosus

## Abstract

**Background:**

Patients with systemic lupus erythematosus (SLE) have substantial unmet medical need. Baricitinib is a Janus kinase (JAK)1 and 2 inhibitor that was shown to have therapeutic benefit in patients with SLE in a phase II clinical trial. The purpose of this study was to evaluate the median change from baseline in conventional serologic biomarkers in subgroups and the overall population of baricitinib-treated patients with SLE, and the SLE Responder Index-4 (SRI-4) response by normalization of anti-dsDNA.

**Methods:**

Data were assessed from the phase II trial I4V-MC-JAHH (NCT02708095). The median change from baseline in anti-dsDNA, IgG, and other conventional serologic markers was evaluated over time in patients who had elevated levels of markers at baseline, and in all patients for IgG. Median change from baseline for baricitinib treatments were compared with placebo. Among patients who were anti-dsDNA positive at baseline, SRI-4 responder rate was compared for those who stayed positive or achieved normal levels by week 24.

**Results:**

Significant decreases of anti-dsDNA antibodies were observed in response to baricitinib 2 mg and 4 mg compared to placebo beginning at weeks 2 (baricitinib 2 mg = − 14.3 IU/mL, placebo = 0.1 IU/mL) and 4 (baricitinib 4 mg = − 17.9 IU/mL, placebo = 0.02 IU/mL), respectively, continuing through week 24 (baricitinib 2 mg = − 29.6 IU/mL, baricitinib 4 mg = − 15.1 IU/mL, placebo=3.0 IU/mL). Significant reductions from baseline of IgG levels were found for baricitinib 4 mg-treated patients compared to placebo at weeks 12 (baricitinib 4 mg = − 0.65 g/L, placebo = 0.09 g/L) and 24 (baricitinib 4 mg = − 0.60 g/L, placebo = − 0.04 g/L). For patients who were anti-dsDNA positive at baseline, no relationship between achieving SRI-4 responder and normalization of anti-dsDNA was observed by week 24.

**Conclusions:**

Baricitinib treatment resulted in a rapid and sustained significant decrease in anti-dsDNA antibodies compared to placebo among those with positive anti-dsDNA antibodies at baseline, as well as a significant decrease in IgG levels in the 4 mg group at weeks 12 and 24. These data suggest that baricitinib may influence B cell activity in SLE. Further studies are needed to evaluate if reductions in anti-dsDNA levels with baricitinib treatment reflect the impact of baricitinib on B cell activity.

**Trial registration:**

NCT02708095.

**Supplementary Information:**

The online version contains supplementary material available at 10.1186/s13075-022-02794-x.

## Background

Systemic lupus erythematosus (SLE) is an autoimmune disease characterized by systemic inflammation, the excessive production of autoantibodies directed at self-antigens, and widespread immune dysregulation [1]. There is evidence that abnormalities in both the innate and adaptive arms of the immune system contribute to disease pathogenesis through a positive feedforward loop, consistent with interferon (IFN) effects [2]. A range of cytokines have been implicated in the etiology of SLE, such as IFNs, B cell activating factor, interleukin (IL)-6, IL-12, IL-17, IL-23, and tumor necrosis factor (TNF) [2, 3]. Dysregulations in cytokine signaling, B cell transcription factors, and B cell-T cell interactions can lead to both the generation of autoreactive B cells and autoantibody production associated with the pathogenesis of SLE [4].

Systemic lupus erythematosus is often characterized by high serological activity, including antibodies against double-stranded DNA (anti-dsDNA). The presence of antibodies that bind to dsDNA have been suggested to contribute to multiple end-organ injuries in SLE [5]. Levels of anti-dsDNA fluctuate with changes in disease activity and, in combination with reduced levels of complement component (C)3 and C4 proteins, are strong indicators of disease flare in patients with SLE [6]. Anti-dsDNA antibodies accompanied by biopsy-proven lupus nephritis (LN) was considered earlier as an independent classification criterion [7] and are also part of the EULAR/ACR 2019 classification for SLE [8, 9] illustrating their key relevance.

The Janus kinase (JAK) family of intracellular, non-receptor tyrosine kinases are important signal transducers associated with many of the key cytokines implicated in immune dysregulation in SLE [1, 10]. Cytokines bind to receptors on the cell membrane and induce phosphorylation of JAKs, and JAKS in turn phosphorylate signal transducer and activator of transcription (STAT) proteins. Phosphorylated STAT proteins then dissociate from the receptor and translocate to the nucleus where they bind to the cytokine response element to activate transcription of specific target genes [11, 12]. Baricitinib is an orally administered, selective, and reversible JAK1/JAK2 inhibitor [13] that has been approved for the treatment of moderate-to-severe active rheumatoid arthritis in adults in over 75 countries including the USA, Japan, and countries in the European Union. Through JAK1/JAK2 inhibition, baricitinib may impact the release of proinflammatory cytokines, such as type I IFNs, IFN-γ, IL-6, IL-12, and IL-23 [10, 14].

In a phase II study of baricitinib in patients with SLE, daily oral baricitinib 4 mg (in addition to standard of care therapy) was superior to placebo with standard of care in improving SLE disease activity at week 24 [14]. However, no significant improvements in least squares (LS) mean change from baseline were observed in the overall population in levels of conventional serologic biomarkers (such as anti-dsDNA antibodies, C3, or C4) for SLE with baricitinib treatment.

The objectives of this analysis were to evaluate the median change from baseline in conventional serologic biomarkers in subgroups (defined in table 1) and in the overall population of baricitinib-treated patients with SLE and to evaluate the SLE Responder Index (SRI-4) response by normalization of anti-dsDNA.

**Table 1:** Summary table of analysis populations for selected autoantibodies and outcomes

Biomarker	Baseline requirement	Placebo (*N* = 105)	Baricitinib 2 mg (*N* = 105)	Baricitinib 4 mg (*N* = 104)
*Anti-dsDNA*	≥30 IU/mL	51 (48.6)	56 (53.3)	53 (51.0)
*IgG*	No requirement	101 (96.2)	99 (94.3)	96 (92.3)
*C3*	<90 mg/dL	30 (28.6)	30 (28.6)	34 (32.7)
*C4*	<10 mg/dL	15 (14.3)	26 (24.8)	15 (14.4)
*Anti-Sm*	≥30 IU/mL	12 (11.47)	7 (6.67)	9 (8.7)
*aCL IgM*	>12 MPL	20 (19.0)	22 (20.9)	22 (21.2)
*aCL IgG*	>14 GPL	10 (9.5)	10 (9.5)	5 (4.8)
*aCL IgA*	>11 APL	4 (3.8)	6 (5.7)	1 (1.0)
*Anti-RNP*	≥30 IU/mL	28 (26.7)	21 (20.0)	28 (27.0)
*Anti-SSA*	>20 IU/mL	35 (33.3)	27 (25.7)	27 (26.0)
*Anti-SSB*	>20 IU/mL	15 (14.3)	15 (14.3)	16 (15.3)

Values are *N*-observed (*N*-observed % of *N*)

*APL/GPL/MPL* arbitrary units for IgA, IgG, and IgM isotypes, respectively, *C* component, *dsDNA* double-stranded deoxyribonucleic acid, *Ig* immunoglobulin, *Sm* Smith, *RNP* ribonucleoproteins, *SS* Sjögren’s syndrome-related antigen, *IU* international unit

## Methods

### Trial design

Patient samples were obtained from the double-blind, multicenter, randomized, placebo-controlled, 24-week phase II clinical trial, I4V-MC-JAHH (NCT02708095) [14]. Eligible patients were aged 18 years or older and had a diagnosis of SLE. At baseline, patients were required to have a positive antinuclear antibody or a positive anti-dsDNA, arthritis, or rash (as defined by Systemic Lupus Erythematosus Disease Activity Index-2000 [SLEDAI-2K]) and a clinical SLEDAI-2K score of 4 or greater. Study drug was added to existing stable background standard of care therapy, which could include glucocorticoids up to 20 mg/day of prednisone or equivalent, a single antimalarial, a single immunosuppressant, and/or non-steroidal anti-inflammatory drugs. Tapering of prednisone or equivalent was permitted from baseline to week 16. Active central nervous system lupus or active severe LN was not permitted.

This study was done in accordance with the ethical principles of the Declaration of Helsinki and Good Clinical Practice guidelines. All investigation sites received approval from the appropriate authorized institutional review board or ethics committee. All patients provided written consent before the study-related procedures were done.

### Randomization and masking

Patients were allocated (1:1:1) using a computer-generated random sequence to baricitinib 2 mg, baricitinib 4 mg, or placebo. Patients were stratified according to disease activity (SLEDAI-2K score <10 or ≥10), anti-dsDNA status (positive or negative), and region (United States of America [USA], Europe, Asia, or rest of the world). Investigators and patients were masked to allocation.

## Outcomes

### Autoantibody expression analysis

Serum samples were analyzed for changes from baseline over time for anti-dsDNA, anti-Smith (Sm), immunoglobulin (Ig)G, and anti-cardiolipin (aCL) antibodies IgM, IgG, and IgA, using INOVA QUANTA Lite SC ELISA® (INOVA Diagnostics, San Diego, CA, USA); and changes from baseline over time for antinuclear ribonucleoprotein (anti-RNP), anti-Sjögren’s syndrome-related (SS) antigen A, and anti-SS antigen B using Semi-Quantitative Multiplex Bead Assay; FIDIS^TM^ (TheraDiag, Paris, France). Changes from baseline over time for complement C3 and C4 were analyzed using the Siemens BNII Nephelometer ^TM^ (Siemens Healthcare Diagnostics, Marburg, Germany). A complete description of baseline requirements and the analysis populations are available in Table 1.

### Systemic lupus erythematosus responder rate analysis

Among patients who were anti-dsDNA positive at baseline, SRI-4 responder rate was compared for those who stayed positive or achieved normal levels (<30 IU/mL) by week 24. The SRI-4 response was defined as a reduction of ≥4 points from baseline in SLEDAI-2K score, no new British Isles Lupus Assessment Group (BILAG) A and ≤1 new BILAG B disease activity scores, and no worsening (defined as an increase of ≥0.3 points (10 mm) from baseline) in the Physician’s Global Assessment of Disease Activity.

### Patient and public involvement statement

Patients were not involved in the research process.

### Statistical analyses

Median changes from baseline for baricitinib 2 mg and baricitinib 4 mg were compared with placebo using a Wilcoxon rank-sum test. In addition, for anti-dsDNA LS mean changes from baseline for baricitinib 2 mg and 4 mg were compared to placebo using mixed-effects model of repeated measures with baseline, region, baseline disease activity (SLEDAI-2K<10 and SLEDAI-2K ≥10), treatment, and treatment-time interaction as variables. Due to the skewness of the laboratory data distribution, evaluating the median change from baseline was more appropriate than the LS mean change from baseline, as the median value is more reflective of the true data. Autoantibody subpopulations used for analysis were based on baseline cut-offs (Table 1). Changes in lab measurements were computed using data from all patients still enrolled in the study at the corresponding time point. For categorical clinical outcomes, such as SRI-4, missing data were imputed using non-responder imputation, and differences between groups were assessed with Fisher’s exact test.

## Results

### Baseline characteristics and disease activity

Most patients were female with a mean age of 43–45 years with a disease duration of 10–12 years (Table 2). Patients randomized into all three treatment arms had comparable anti-dsDNA, IgG, C3, and C4 at baseline (Table 2).

**Table 2:** Baseline characteristics and disease activity

	Placebo (*N* = 105)	Baricitinib 2 mg (*N* = 105)	Baricitinib 4 mg (*N* = 104)
Age, years, mean (SD)	44.9 (12.8)	43.2 (11.0)	45.0 (12.4)
Female, *n* (%)	99 (94.3)	96 (91.4)	99 (95.2)
Time since onset of SLE symptoms, years, mean (SD)	9.7 (7.7)	11.8 (9.1)	11.5 (10.3)
SLEDAI-2K score, mean (SD)	8.9 (2.9)	8.8 (3.4)	9.0 (3.3)
≥1 A or ≥2 B BILAG scores, *n* (%)	62 (59.0)	56 (53.3)	69 (66.3)
Physician’s Global Assessment score^a^, mean (SD)	1.5 (0.5)	1.5 (0.5)	1.6 (0.5)
Positive anti-dsDNA, *n* (%)	51 (48.6)	56 (53.3)	53 (51.0)
Anti-dsDNA, IU/mL, median (range)^b^	125.8 (33, 999)	139.8 (35, 1167)	128.4 (31, 1864)
Immunoglobulin G, g/L, median (range)^c^	13.4 (5.2–53.4)	13.5 (6.3–26.1)	13.0 (6.9–40.6)
Complement C3, g/L, median (range)	1.0 (0.3–1.7)	1.0 (0.4–1.7)	1.1 (0.26–2.0)
Complement C4, g/L, median (range)	0.2 (0.0–0.5)	0.2 (0.0–0.4)	0.2 (0.0–0.9)

Data are presented as mean (standard deviation) unless stated otherwise

^a^ Scores range from 0 to 3 on a visual analogue scale with higher values indicating more severe disease

^b^ Reported for patients with anti-dsDNA ≥30 IU/mL at baseline

^c^ Placebo, *n* = 101; baricitinib 2 mg, *n* = 99; baricitinib 4 mg, *n* = 96)

*BILAG* British Isles Lupus Assessment Group, *C* component, *dsDNA* double-stranded deoxyribonucleic acid, *N* number of patients, *n* number of patients in a subgroup, *SLE* systemic lupus erythematosus, *SLEDAI-2K* Systemic Lupus Erythematosus Disease Activity Index-2000

### Median and LS mean change from baseline in anti-dsDNA

**Fig. 1:**
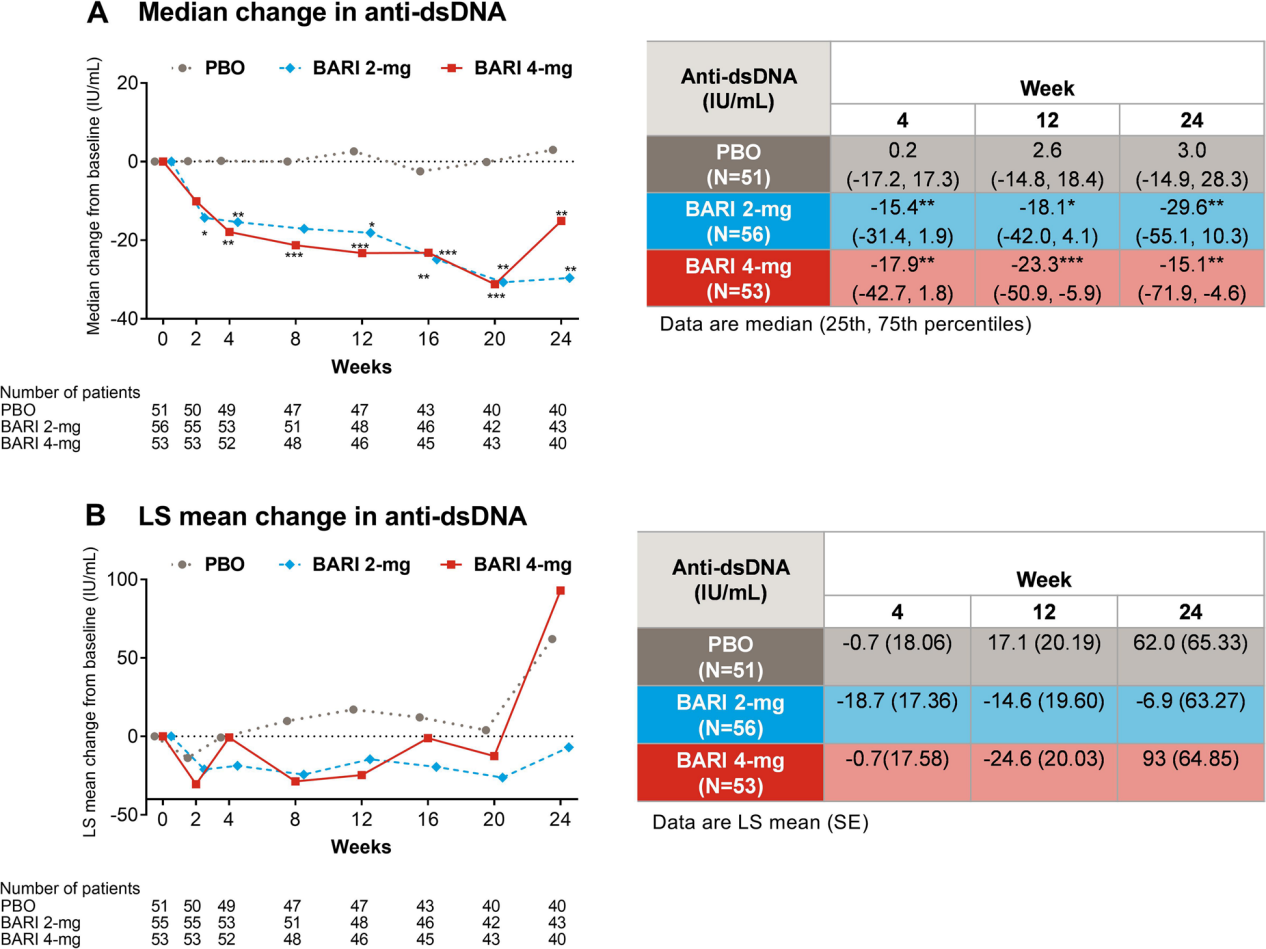
Median (**a**) and LS mean (**b**) change from baseline in anti-dsDNA (IU/mL). Data were assessed for significance in patients who were anti-dsDNA positive (≥30 IU/mL) at baseline. *p ≤0.05, **p≤0.01, ***p≤0.001 for BARI vs PBO. BARI, baricitinib; LS mean, least squares mean; PBO, placebo; dsDNA, double-stranded deoxyribonucleic acid

In the subgroup of patients with increased anti-dsDNA at baseline, significant decreases in median anti-dsDNA antibody levels were observed for baricitinib 2 mg and baricitinib 4 mg compared with placebo beginning at week 2 (baricitinib 2 mg = − 14.3 IU/mL, placebo = 0.1 IU/mL, *p* = 0.028) and week 4 (baricitinib 4 mg = − 17.9 IU/mL, placebo = 0.2 IU/mL, *p* = 0.003) respectively. These decreases were sustained through week 24 (baricitinib 2 mg = − 29.6 IU/mL, baricitinib 4 mg = − 15.1 IU/mL, placebo = 3.0 IU/mL) (Fig. 1a). In the same subgroup, no significant changes were reported in LS mean anti-dsDNA antibody levels in response to baricitinib 2 mg or baricitinib 4 mg compared to placebo at any time point measured (Fig. 1b).

### Median change in conventional serologic markers

**Fig. 2:**
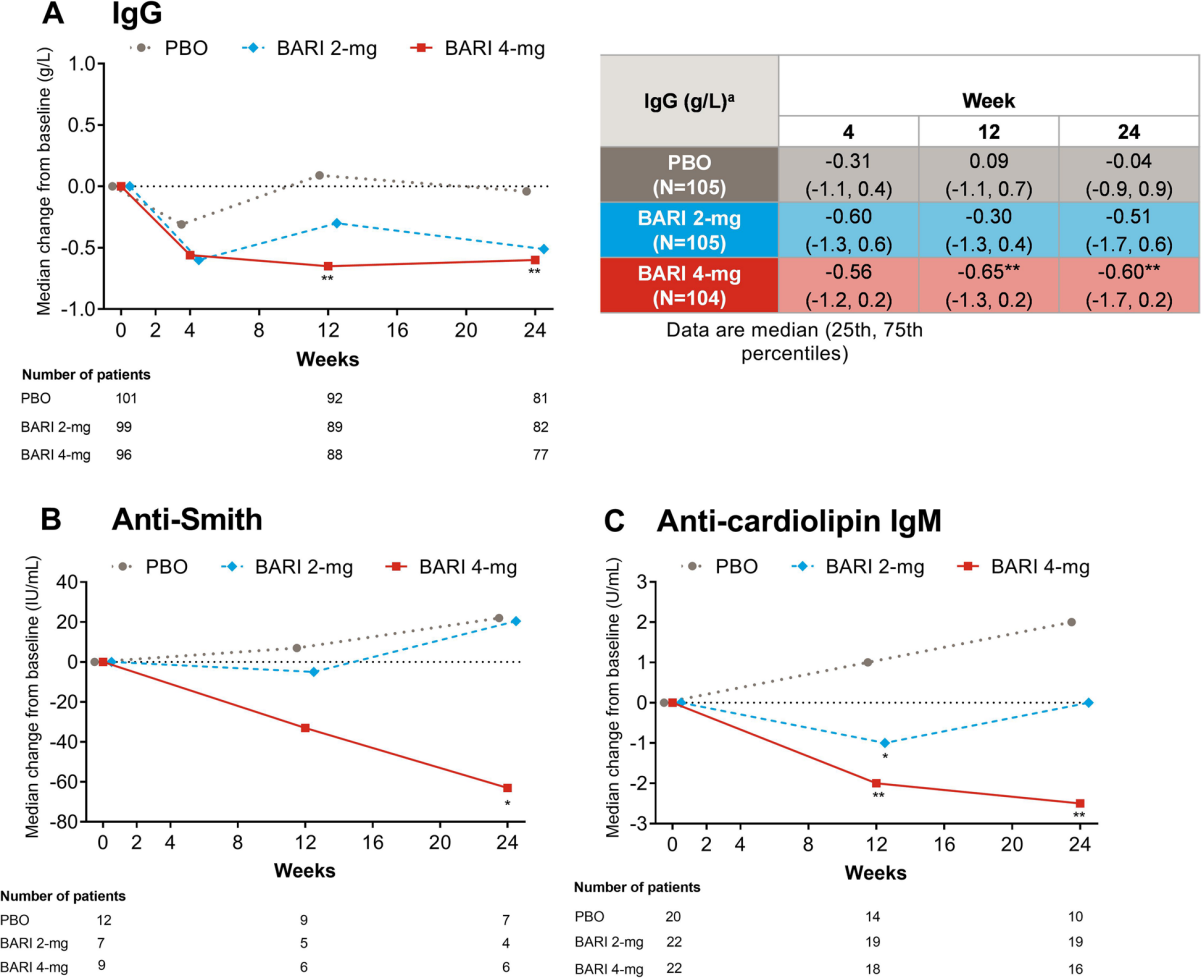
Median change from baseline in IgG (**a**), anti-Smith (**b**), and anti-cardiolipin IgM (**c**). **A** Data were assessed for significance in all patients. **B** Data were assessed for significance in patients with anti-Smith ≥30 IU/mL. **C** Data were assessed for significance in patients with anti-cardiolipin IgM>12 MPL at baseline. *p ≤ 0.05, **p ≤ 0.01 for BARI vs. PBO. BARI, baricitinib; Ig, immunoglobulin; PBO, placebo; MPL, IgM phospholipid units

In the intent-to-treat population, treatment with baricitinib 4 mg resulted in a significant decrease in the median IgG levels compared to placebo from week 12 (baricitinib 4 mg = − 0.65 g/L, placebo = 0.09 g/L, *p* = 0.004, Fig. 2a) through week 24 (baricitinib 4 mg = − 0.60 g/L, placebo = − 0.04 g/L, *p* = 0.003, Fig. 2a). A numerical decrease was also observed in response to baricitinib 2 mg.

In the subgroup of patients with increased anti-Sm at baseline, median anti-Sm levels were numerically decreased in response to baricitinib 4 mg at week 12 compared to placebo (baricitinib 4 mg = − 33.0 IU/mL, placebo = 7.0 IU/mL, Fig. 2b) and a significant difference was reported at week 24 (baricitinib 4 mg = − 63.0 IU/mL, placebo = 22.0 IU/mL, *p* = 0.017, Fig. 2b). Baricitinib 2 mg did not influence anti-Sm levels compared to placebo.

In the subgroup of patients with increased aCL IgM at baseline, median aCL IgM was significantly decreased by baricitinib 2 mg (− 1.0 U/mL, *p* = 0.034) and 4 mg (− 2.0 U/mL, *p* = 0.007) compared to placebo (1.0 U/mL) at week 12 (Fig. 2c). At week 24, baricitinib 4 mg (− 2.50 U/mL) treatment resulted in a sustained statistically significant reduction compared to placebo (2.0 U/mL, *p* = 0.011, Fig. 2c).

### Median change from baseline in other autoantibodies

In the defined subgroups, no significant changes in median values from baseline were observed in aCL IgG, aCL IgA, anti-RNP, or anti-SSA and anti-SSB with baricitinib 2 mg or 4 mg treatment at any timepoint up to week 24 (supplementary material, Fig. S2).

### Median change in C3 and C4

**Fig. 3:**
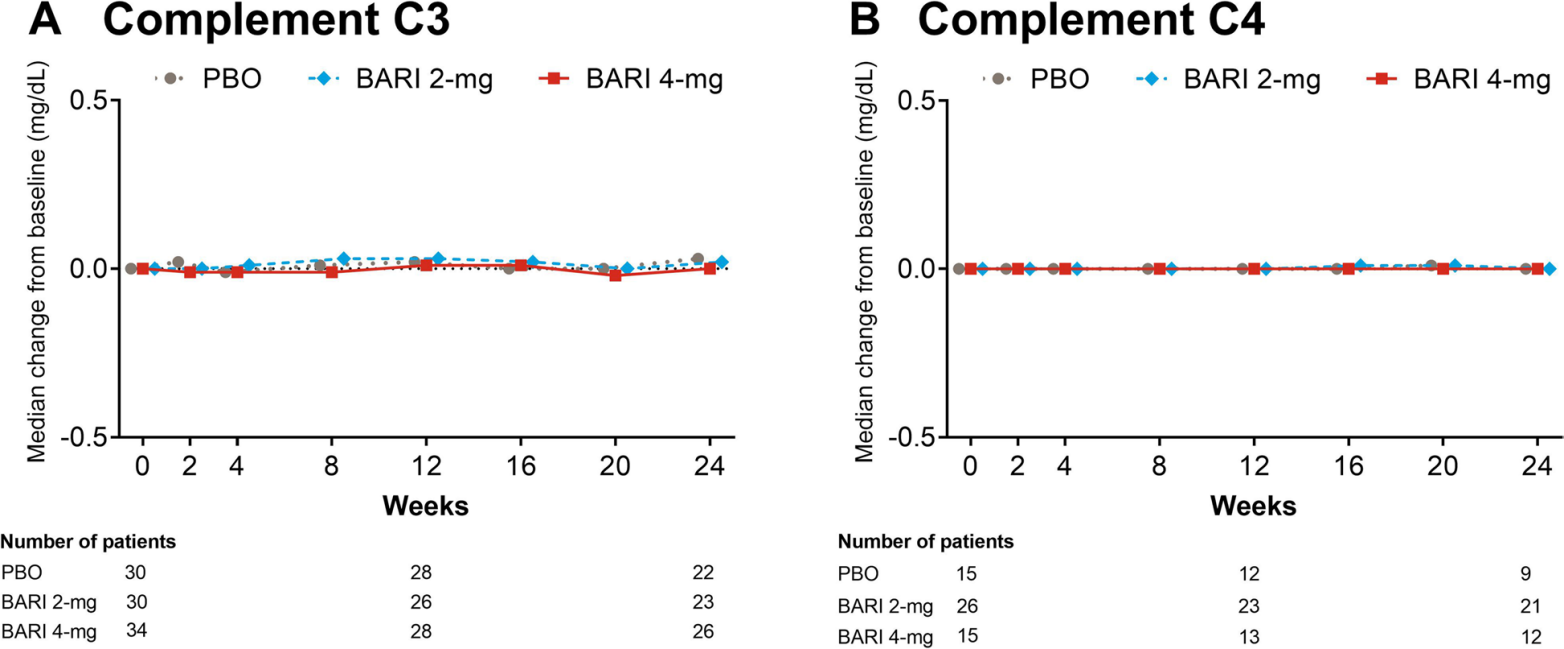
Median change from baseline in C3 (**a**) and C4 (**b**). Data were assessed for significance in patients with C3<90 mg/dL, or C4<10 mg/dL at baseline. BARI, baricitinib; C, component; PBO, placebo

In the defined subgroups, no significant differences in median change of C3 and C4 from baseline were observed for baricitinib 2 mg or baricitinib 4 mg treatment at any time point up to week 24 (Fig. 3).

### Normalization of anti-dsDNA levels and SRI-4 response

**Fig. 4:**
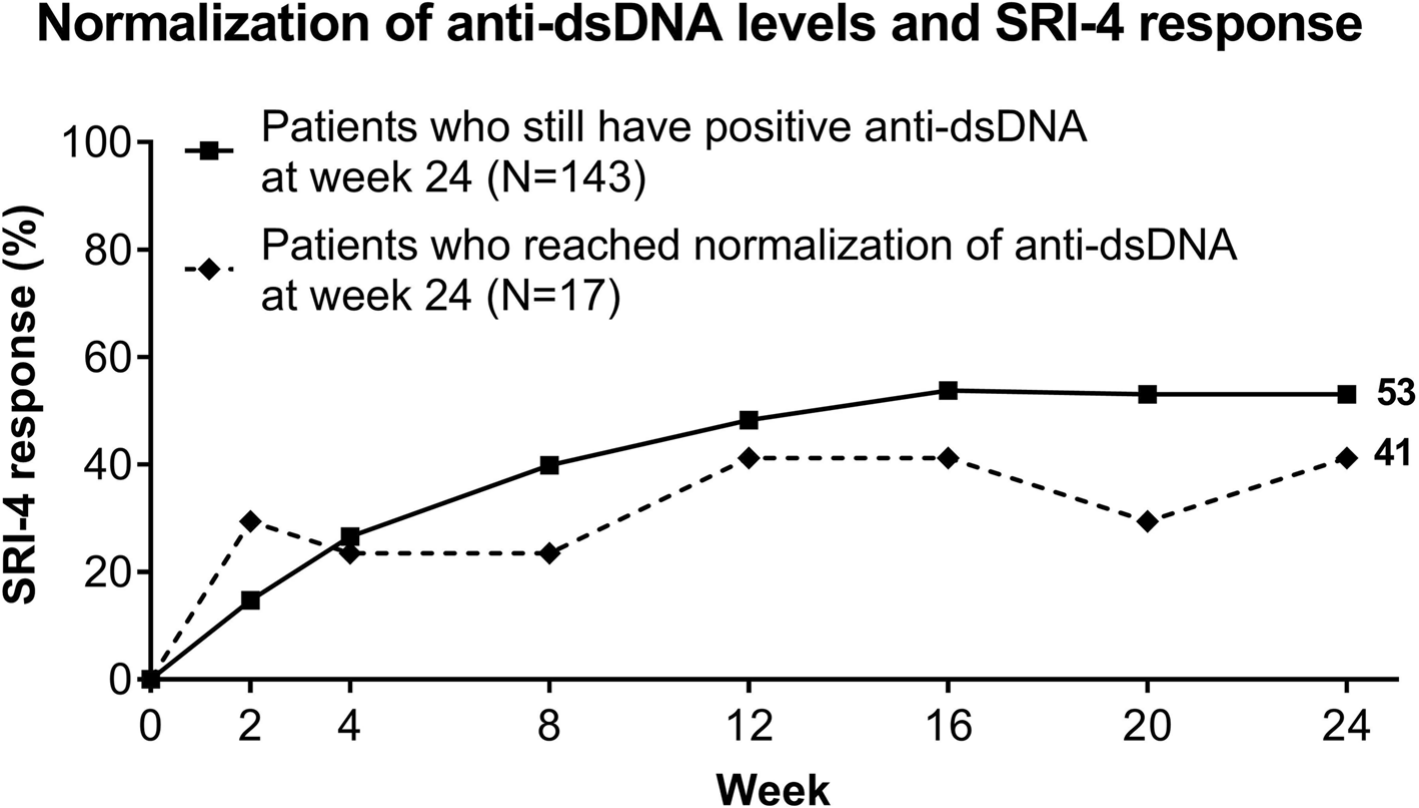
Normalization (defined by a reduction of anti-dsDNA levels to <30 IU/mL) of anti-dsDNA levels and SRI-4 response. Data were assessed for significance for all patients with anti-dsDNA ≥30 IU/mL at baseline, irrespective of treatment assignment. dsDNA, double-stranded deoxyribonucleic acid; N, number of patients; SLE, systemic lupus erythematosus SRI-4, SLE Responder Index-4

The SRI-4 response rate was assessed between patients who remained anti-dsDNA positive post-baseline, and those who achieved normal anti-dsDNA levels. There was no statistically significant difference in SRI-4 responder rate between those who stayed positive, defined as patients with ≥30 IU/mL (*N* = 143), or those who achieved normalization, defined as patients with <30 IU/mL (*N* = 17), at any timepoint up to week 24, irrespective of treatment with baricitinib 2 mg, 4 mg, or placebo (Fig. 4).

## Discussion

The goal of this study was to evaluate the effects of the JAK1/JAK2 inhibitor baricitinib on the median change from baseline in conventional serologic biomarkers in subgroups and the overall population of SLE patients and the SRI-4 response by normalization of anti-dsDNA in a phase II trial.

There are several limitations to the conclusions that can be drawn from this study. While the results support the use of baricitinib in the treatment of SLE, the short timeframe of 24 weeks limits the ability to assess longer-term outcomes. In addition, the small size of the study cohort in this phase II trial is a limiting factor when interpreting the analysis. Data from two larger phase III trials (NCT03616912 and NCT03616964) will further delineate the current findings.

Treatment with baricitinib resulted in a rapid and sustained, statistically significant decrease in anti-dsDNA antibodies compared with placebo in SLE patients positive for anti-dsDNA antibodies at baseline. Treatment with baricitinib 4 mg also resulted in a statistically significant decrease in IgG and aCL IgM levels at weeks 12 and 24 and anti-Sm at week 24 compared with placebo. Increased anti-Sm levels are associated with several clinical symptoms of SLE such as renal, neurologic, and hematologic manifestations [15]. The decrease in anti-Sm seen in this study could represent reduced disease activity but may also reflect decreased immunoglobulin levels. There was no measurable relationship between SRI-4 response rate and anti-dsDNA levels, irrespective of treatment with baricitinib or placebo. This may be due to the limited sample size of the study or may indicate that there is no relationship between anti-dsDNA levels and SRI-4 response rates.

Cytokines such as IL-6, IL-10, IL-12, and IFNs play critical roles in B cell hyperactivity and differentiation, autoantibody production, and the immunopathology of SLE [16–19]. IL-6, IL-10, and IFNs have been shown to positively correlate with measures of disease activity, such as SLEDAI, and with autoantibody levels in SLE [16, 18, 20–23]. Recently, microarray analysis on serum samples from this study cohort found that baricitinib 4 mg decreased IL-12p40 and IL-6 (known as a potent stimulator of B cells) at week 12 [24]. Thus, through its inhibition of JAK1/JAK2 signaling, baricitinib may exert downstream effects on B cell activity, thereby mediating its effect on clinical symptoms in SLE [14, 25–27].

In the defined patient subgroups, baricitinib treatment combined with standard of care led to significant reductions in autoantibodies including anti-dsDNA, as well as IgG levels. These observed reductions are probably indicative of reduced B cell activity with baricitinib treatment. What is less clear is if this translates into a therapeutic effect or if this is simply an epiphenomenon due to reduced antibody production, as evidenced by decreasing IgG levels.

The titer of anti-dsDNA antibodies is a clinically useful tool to measure disease activity and predict flares in patients with SLE. Anti-dsDNA antibodies can be positive for at least 2 years before a diagnosis of SLE, and an increase in serum levels of anti-dsDNA antibodies is a predictor of symptom flares in patients with SLE [28]. In LN, anti-dsDNA antibodies form immune complexes through interaction with renal antigens and are present in nearly 80% of patients with LN [29]. Stimulating human mesangial cells with anti-dsDNA antibodies promotes the production of proinflammatory cytokines, such as TNF, IL-1β, and IL-6 [30, 31].

Anti-dsDNA can also contribute to LN severity through upregulation of renal fibrosis. Anti-dsDNA IgG isotype can downregulate suppressor of cytokine signaling 1 and activate JAK/STAT 1 signals, which influence the expression of profibrotic genes, such as transforming growth factor beta 1, connective tissue growth factor, and platelet-derived growth factor B [32, 33].

Anti-dsDNA has also been shown to contribute to inflammation of the skin through deposition of an immune complex at the dermoepidermal junction and to contribute to neuropsychiatric complications through cross-reaction with anti-N-methyl-D-aspartate receptor (NMDAR), and NMDARs on neurons, in patients with SLE [34, 35].

In this phase II study, baricitinib 2 mg and 4 mg reduced median anti-dsDNA levels significantly compared to placebo in the subpopulation of patients with high anti-dsDNA at baseline. Given the evidence showing anti-dsDNA involvement in disease activity in SLE, reductions in anti-dsDNA levels with baricitinib treatment could potentially be therapeutic in patients with SLE, a marker of reduced disease activity or the reduction could be merely coincidental, reflecting a decreased antibody production in a non-specific manner. In this study, there was no measurable effect of decreased anti-dsDNA on SRI-4 response rates, irrespective of treatment with baricitinib or placebo. This may have been due to the limited sample size used in this study or may indicate that there is no relationship between anti-dsDNA levels and SRI-4 response rates in the population analyzed. Results presented here should be interpreted with caution, validation by subsequent studies is mandated to evaluate if reductions in anti-dsDNA levels with baricitinib treatment are coincidental or reflect the impact of baricitinib on B cell activity.

## Conclusions

As part of the inhibition of cytokine signaling through JAK1 and JAK2 in patients with SLE, the mechanism of action of baricitinib may be mediated partially through the downstream inhibition of autoreactive B cell activation, as manifest by reduction in IgG and autoantibodies including anti-dsDNA.

## Supplementary Information

**Additional file 1: Figure S1.** Trial profile participant flow chart.

**Additional file 2: Figure S2.** Median change from baseline in aCL IgA, aCL IgG, anti-SSA, Anti-SSB, anti-RNP.

## Data Availability

The datasets generated during and/or analyzed during the current study are available from the corresponding author on reasonable request.

## Abbreviations

aCL: Anti-cardiolipin
dsDNA: Double-stranded deoxynucleic acid
anti-RNP: Antinuclear ribonucleoprotein
BILAG: British Isles lupus assessment group
C: Component
IFN: Interferon
IgG: Immunoglobulin
IL: Interleukin
IU: International unit
JAK: Janus kinase
LN: Lupus nephritis
LS: Least squares
*n*: Number of patients in a subgroup
*N*: Number of patients
NMDAR: N-methyl-D-aspartate receptor
SD: Standard deviation
SLE: Systemic lupus erythematosus
SLEDAI-2K: SLE Disease Activity Index-2000
Sm: Smith
SRI-4: SLE Responder Index-4
SS: Sjögren’s syndrome-related
STAT: Signal transducer and activator of transcription
TNF: Tumor necrosis factor
USA: United States of America
