# Supplementary figures and images for: Baricitinib decreases anti-dsDNA in patients with systemic lupus erythematosus: results from a phase II double-blind, randomized, placebo-controlled trial
Source: Arthritis Res Ther. 2022 May 16;24:112. doi: 10.1186/s13075-022-02794-x (PMC9109322; doi:10.1186/s13075-022-02794-x)

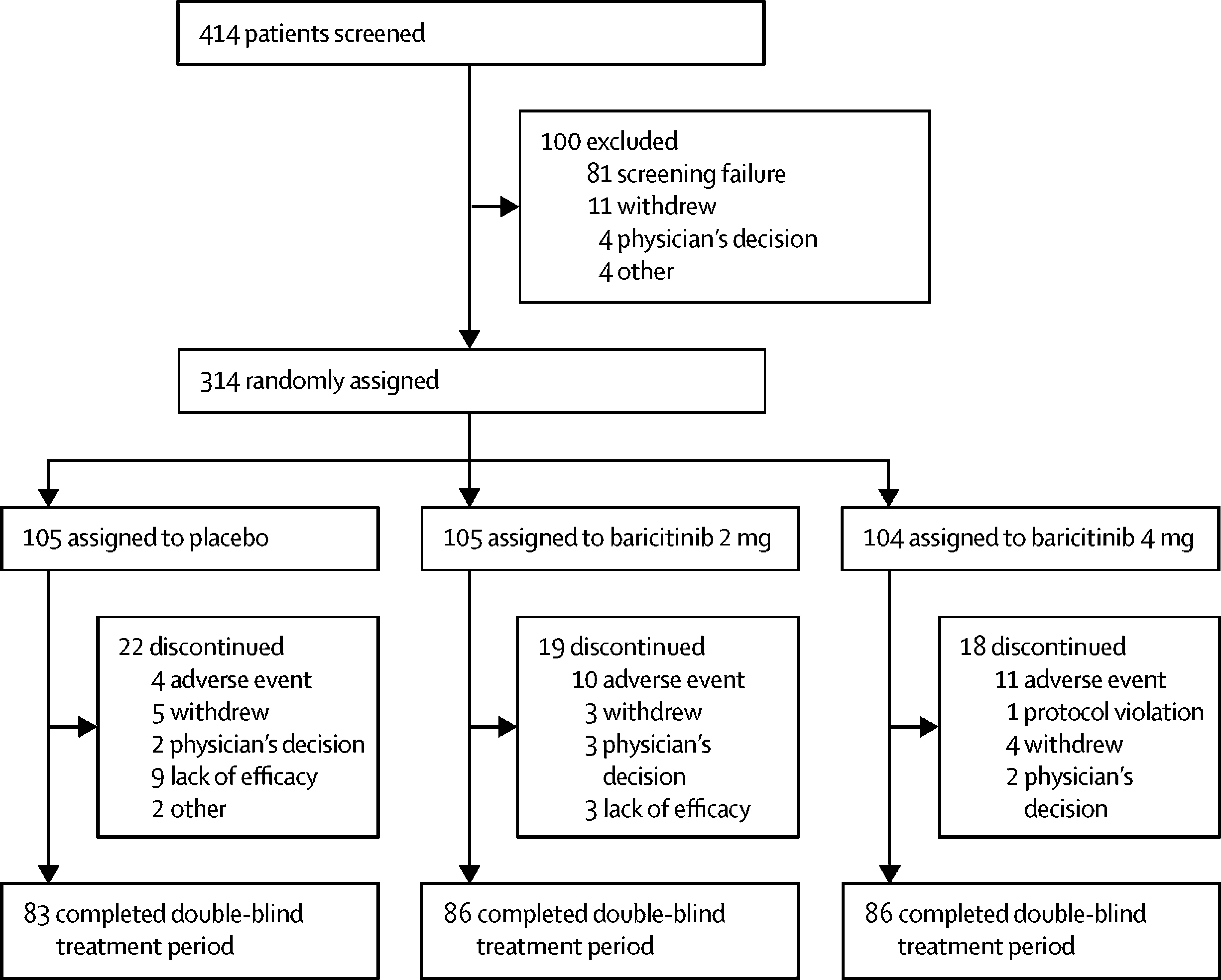

Supplement: Supplementary file 1 — Additional file 1: Figure S1. Trial profile participant flow chart. [file 13075_2022_2794_MOESM1_ESM.jpg]

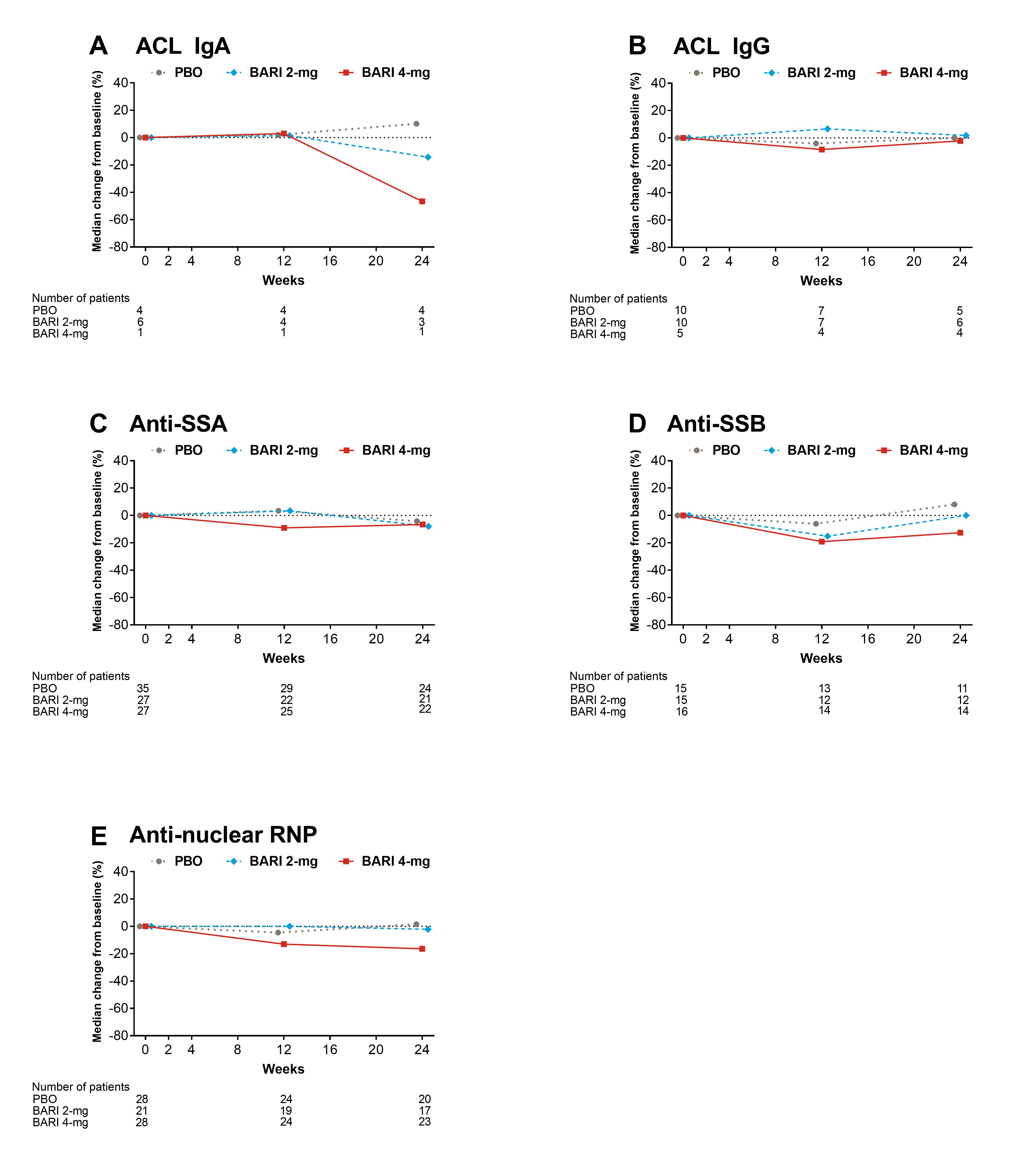

Supplement: Supplementary file 2 — Additional file 2: Figure S2. Median change from baseline in aCL IgA, aCL IgG, anti-SSA, Anti-SSB, anti-RNP. [file 13075_2022_2794_MOESM2_ESM.jpg]
